# Increased Production of α-Linolenic Acid in Soybean Seeds by Overexpression of Lesquerella *FAD3-1*

**DOI:** 10.3389/fpls.2019.01812

**Published:** 2020-01-31

**Authors:** Wan Woo Yeom, Hye Jeong Kim, Kyeong-Ryeol Lee, Hyun Suk Cho, Jin-Young Kim, Ho Won Jung, Seon-Woo Oh, Sang Eun Jun, Hyun Uk Kim, Young-Soo Chung

**Affiliations:** ^1^ Department of Molecular Genetics, College of Natural Resources and Life Science, Dong-A University, Busan, South Korea; ^2^ Department of Agricultural Biotechnology, National Institute of Agricultural Science, Rural Development Administration, Jeonju, South Korea; ^3^ Biosafety Division, National Institute of Agricultural Science, Rural Development Administration, Jeonju, South Korea; ^4^ Department of Bioindustry and Bioresource Engineering, Plant Engineering Research Institute, Sejong University, Seoul, South Korea

**Keywords:** ALA: alpha-linolenic acid, FAME: fatty acid methyl ester, NT: non-transgenic, Pβ: β-conglycinin promoter, Pphas: phaseolin promoter

## Abstract

Soybean is a major crop that is used as a source of vegetable oil for human use. To develop transgenic soybean with high α-linolenic acid (ALA; 18:3) content, the *FAD3-1* gene isolated from lesquerella (*Physaria fendleri*) was used to construct vectors with two different seed-specific promoters, soybean β-conglycinin (Pβ-con) and kidney bean phaseolin (Pphas), and one constitutive cauliflower mosaic virus 35S promoter (P35S). The corresponding vectors were used for *Agrobacterium*-mediated transformation of imbibed mature half seeds. The transformation efficiency was approximately 2%, 1%, and 3% and 21, 7, and 17 transgenic plants were produced, respectively. T-DNA insertion and expression of the transgene were confirmed from most of the transgenic plants by polymerase chain reaction (PCR), quantitative real-time PCR (qPCR), reverse transcription PCR (RT-PCR), and Southern blot analysis. The fatty acid composition of soybean seeds was analyzed by gas chromatography. The 18:3 content in the transgenic generation T_1_ seeds was increased 7-fold in Pβ-con:*PfFAD3-1*, 4-fold in Pphas : *PfFAD3-1*, and 1.6-fold in P35S:*PfFAD3-1* compared to the 18:3 content in soybean “Kwangankong”. The increased content of 18:3 in the Pβ-con:*PfFAD3-1* soybean (T_1_) resulted in a 52.6% increase in total fatty acids, with a larger decrease in 18:1 content than 18:2 content. The increase in 18:3 content was also maintained and reached 42% in the Pphas : *PfFAD3-1* transgenic generation T_2_. Investigations of the agronomic traits of 12 Pβ-con:*PfFAD3-1* transgenic lines (T_1_) revealed that plant height, number of branches, nodes, pods, total seeds, and total seed weight were significantly higher in several transgenic lines than those in non-transgenic soybean. Especially, an increase in seed size was observed upon expression of the *PfFAD3-1* gene with the β-conglycinin promoter, and 6%–14% higher seed lengths were measured from the transgenic lines.

## Introduction

Soybean (*Glycine max* (L.) Merr.) is an important crop that serves as a significant source of oil (~20%) and protein (~40%). The agricultural importance of soybean has been recognized owing to its various beneficial effects on human health (Oksman-Caldentey and Hiltunen, 1996; Zeng et al., 2004; Manavalan et al., 2009; John et al., 2016). Given its amenability to genetic transformation, soybean has been subjected to gene transfer. Soybean is the world's largest genetically modified crop because of its applications in food, industrial, and pharmaceutical products (Li et al., 2017; Chen et al., 2018). Initially, research on biotech soybean focused on agronomic traits for securing yields. There has been great interest in the development of biotech crops with value-added traits to improve nutritional value and industrial applications (Homrich et al., 2012). Increasing interest in the production of functional crops has propelled the development of soybean crops with specific new nutrients and increased functionality. Trials to produce or increase the levels of functional compounds, such as isoflavone, β-carotene, and syringin, were conducted in soybean calluses and seeds using *Agrobacterium*-mediated transformation (Jiang et al., 2010; Kim et al., 2012; Kwon et al., 2017).

Increasing the oil and protein content of soybean seed has been a task for breeders to meet the demand of the rapidly growing human population and the rising concern of food shortage (El-Hamidi and Zaher, 2018). Both quantity and quality are important factors to improve soybean oil production (Al Amin et al., 2019; Kanai et al., 2019). The fatty acids in soybean seed oil include palmitic acid (11%), stearic acid (4%), oleic acid (23%), linoleic acid (LA) (54%), and α-linolenic acid (ALA) (8%). At present, soybean oil is mainly used for frying; hence, molecular breeding is being utilized to reduce the production of *trans* fat from polyunsaturated fatty acids at high temperatures. The main goal of breeding was to increase the content of oleic acid and reduce the LA and ALA levels (Flores et al., 2008; Pham et al., 2012; Yang et al., 2018; Al Amin et al., 2019). However, some contradictory reports have mentioned the benefits of high LA and ALA to human health (Rao et al., 2008; Amjad Khan et al., 2017). With the increase in market demand for functional food materials and industrial feedstock, there is a growing need to develop a soybean variety that produces high levels of functional omega-3 fatty acids. ALA, an omega-3 fatty acid in plants, is essential for humans and is obtained only from the diet. ALA is converted to eicosapentaenoic acid (EPA) and docosahexaenoic acid (DHA) in the human body. In addition, ALA is used as an environmentally friendly coating material such as linoleum used for floor covering (Jhala and Hall, 2010). Omega-3 fatty acids are critical for the human biological system and particularly helpful for the prevention of cardiovascular diseases (Psota et al., 2006; Khan et al., 2017). However, the content of omega-3 fatty acids in soybean seed is ~8%, which is relatively lower than that in other plants rich in omega-3. The recommended intake ratio of omega-6/omega-3 in healthy diets varies from 5:1 to 1:1 (Simopoulos, 2000). Therefore, it is desirable to increase the level of omega-3 ALA and lower the content of omega-6 LA in soybean. The enzyme involved in the synthesis of ALA is microsomal omega-3 fatty acid desaturase 3 (FAD3), which forms a double bond between the 15th and 16th carbon atoms of LA to synthesize ALA (Arondel et al., 1992). Soybean possesses four *FAD3* genes, three of which (*GmFAD3-1a*, *GmFAD3-1b*, and *GmFAD3-2a*) are expressed in the seeds and control ALA content in the seed oil (Bilyeu et al., 2003; Anai et al., 2005).

We had previously cloned two genes, *PfFAD3-1* and *PfFAD3-2*, from lesquerella (*Physaria fendleri*), a new oil crop that produces an industrially useful hydroxy fatty acid, lesquerolic acid (20:1-OH). Introduction of the *PfFAD3-1* gene into *FAD3*-deficient *Arabidopsis* resulted in an increase in the ALA content from 1.6% to 30% (Lee et al., 2019). Although the soybean genome contains three *FAD3* genes that are expressed in the seeds, ALA content in the seeds is low (8%). The reason could be that soybean FAD3s have low enzyme activity or this enzymatic activity is impacted by feedback inhibition. Thus, we decided to use heterologous *PfFAD3-1*. In the present study, *PfFAD3-1* was transformed into soybean to develop healthy functional soybean varieties with increased omega-3 ALA content.

## Materials and Methods

### Construction of Three Types of *PfFAD3-1* Expression Vectors for Soybean Transformation

**Figure 1:**
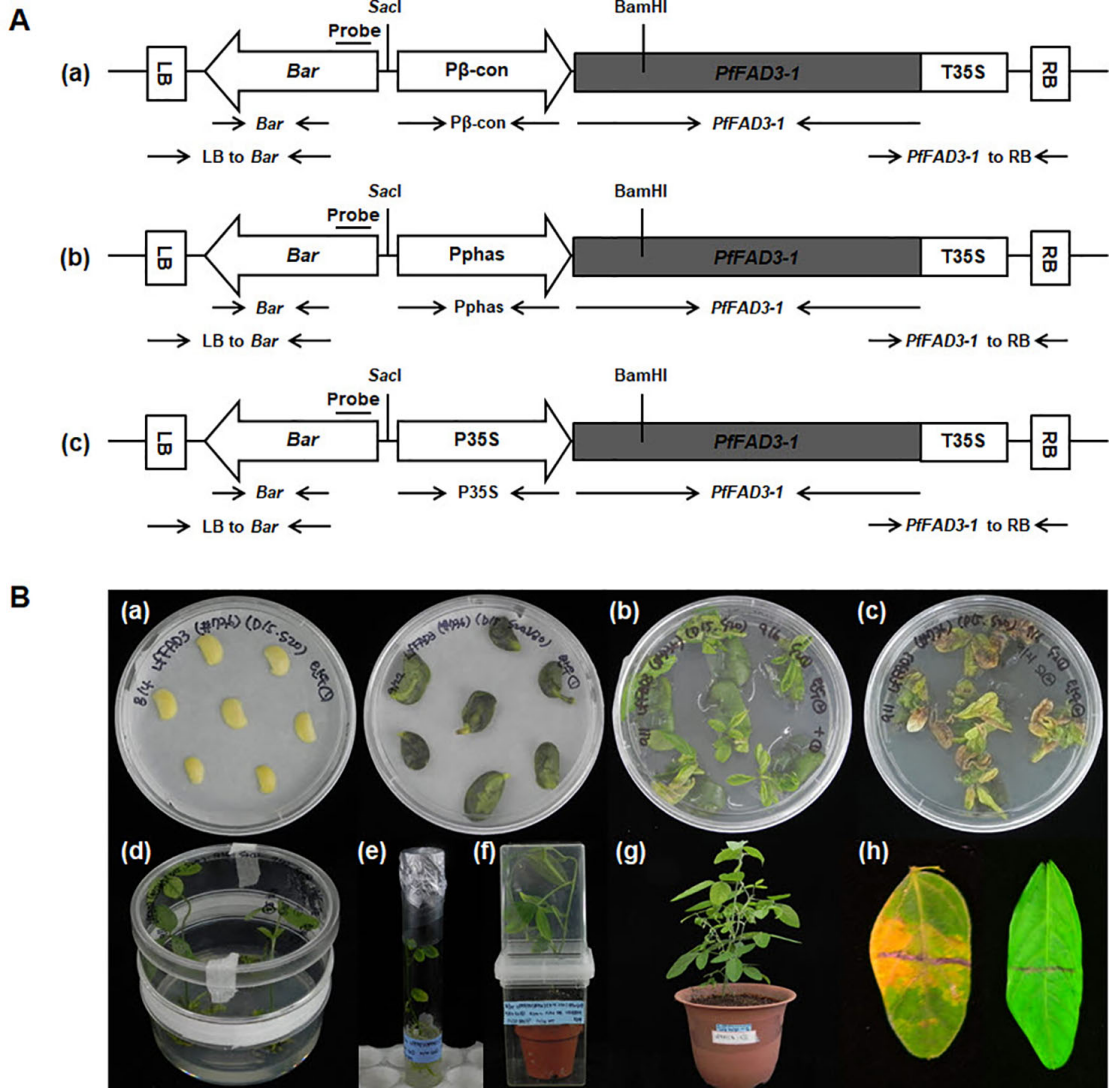
Production of soybean transgenic plants with the *PfFAD3-1* gene *via Agrobacterium*-mediated transformation. **(A)** Vectors used for soybean transformation. The amplified *PfFAD3-1* (1,164 bp size) was subcloned into the pB2GW7.0 vector carrying the β-conglycinin promoter (548 bp size) (a), phaseolin promoter (1,543 bp size) (b), and CaMV-35S promoter (953 bp size) (c). LB/RB, left/right T-DNA border; Pβ-con, soybean seed-specific β-conglycinin promoter; Pphas, seed-specific phaseolin promoter derived from *Phaseolus vulgaris*; P35S/T35S, cauliflower mosaic virus (CaMV) 35S promoter/terminator; *Bar*, coding region of the DL-phosphinothricin acetyltransferase gene that confers resistance to the herbicide glufosinate. *Sac*I and *Bam*HI restriction enzymes sites are marked. **(B)** Production of *PfFAD3-1* soybean transgenic plants. (a) Half-seed explants after inoculation (left) and at 5 days after inoculation (right). (b) Shoot induction on SIM without PPT selection for 14 days. (c) Shoot induction on SIM with 10 mg/L PPT for another 14 days. (d) Shoot elongation on SEM with 5 mg/L PPT. (e) Root formation on RM. (f) Acclimation of a putative transgenic plant in a small pot. (g) Transgenic plant (T_0_) cultivated in a large pot in a greenhouse. (h) Non-transgenic plant leaf was sensitive (left), whereas the leaf from the transgenic plant was resistant (right) 5 days after herbicide (100 mg/L PPT) leaf painting.

The gene *PfFAD3-1* (Accession No. MF611845) was amplified using the first-strand cDNA of the total RNA from the developing seeds of the lesquerella germplasm line WCL-LY2 (Dierig et al., 2001) as a template for polymerase chain reaction (PCR) using *PfFAD3-1* forward primer (5ʹ-CACCATGGTGGTTGCTATGGACA-3ʹ) and reverse primer (5ʹ-TAATTGATTTTAGATTTGTCAG-3ʹ). The resulting PCR product was subcloned into the vector pENTR/D-TOPO (Invitrogen, USA) and, then, recombined into the three desired destination vectors, pB2GW7.0-Pβ-conglycinin (pB2GW7.0 vector carrying a soybean seed-specific β-conglycinin promoter), pB2GW7.0-P35S (pB2GW7.0 vector with the CaMV 35S promoter), and pB2GW7.0-Pphaseolin (pB2GW7.0 vector carrying a seed-specific phaseolin promoter derived from *Phaseolus vulgaris*) (VIB-Ghent University, Ghent, Belgium) using the Gateway cloning method. These three constructed plasmids (Pβ-con:*PfFAD3-1*, Pphas : *PfFAD3-1*, and P35S:*PfFAD3-1*; **Figure 1A**) were transformed into *Agrobacterium tumefaciens* EHA105 (Karimi et al., 2002) as per the protocol described by Kim et al. (2013; 2016).

### Selection of *PfFAD3-1* Transformed Soybeans

Mature soybean seeds of the Korean cultivar (*Glycine max* L. “Kwangankong”) were used, following the half-seed method for soybean transformation (Paz et al., 2006) with modifications. Six batches of transformation were carried out with 100–120 seeds per batch. Sterilized soybean seeds were soaked in distilled water for 12 h at 24°C. The *Agrobacterium* culture was centrifuged for 15 min at 20°C, 7,000 rpm. The cell pellet was resuspended in 15 ml liquid CCM (Co-Cultivation Medium) containing 0.32 g·L^−1^ Gamborg B5 salt with B5 vitamins, 4.26 g·L^−1^ 2-(N-morpholino)ethanesulfonic acid (MES), 3% sucrose, 1.67 mg·L^–1^ 6-benzylaminopurine, 0.25 mg·L^−1^ gibberellic acid, 0.2 mM acetosyringone, 3.3 mM L-cysteine, 1.0 mM sodium thiosulfate, and 1.0 mM dithiothreitol (DTT), pH 5.4. The cotyledons were separated by a longitudinal cut, and the seed coat was removed. The embryonic axis found at the junction between the hypocotyl and the cotyledon was excised to obtain the half-seed explant. The junction area was wounded eight times using a No. 11 scalpel blade (Feather, Japan) and, then, treated with the 15 ml liquid CCM containg Agrobacterium suspension as a dipping solution**. Approximately 50 half-seed explants were immersed in 15 ml *Agrobacterium* suspension and inoculated for 30 min at room temperature. After inoculation, the explants were placed adaxial side down on CCM containing 5 g·L^−1^ agar overlaid with filter paper (Advantec, Japan). The co-cultivation was continued for 5 d at 24°C under an 18-h photoperiod. After 5 d of co-cultivation, the half-seed explants were briefly washed in liquid SIM (shoot induction medium) containing 3.2 g·L^−1^ B5 salt with B5 vitamins, 0.6 g·L^−1^ MES, 3% sucrose, 1.67 mg·L^−1^ +6-benzyladenine (BA), 250 mg·L^−1^ cefotaxime, 100 mg·L^−1^ ticarcillin, and 50 mg·L^−1^ vancomycin for 10 min. The explants were embedded in SIM containing 5.4 g·L^−1^ agar with the flat side up. Shoot induction was carried out for 14 d at 24°C under an 18-h photoperiod in a tissue culture room. Explants were also transferred to fresh SIM containing 10 mg·L^−1^ DL-phosphinothricin (PPT) for 14 d. After 4 weeks of shoot induction, the explants were transferred to SEM (shoot elongation medium) containing 4.4 g·L^−1^ MS salt with B5 vitamins, 0.6 g·L^−1^ MES, 3% sucrose, 5.4 g·L^−1^ agar, 0.5 mg·L^−1^ gibberellic acid, 50 mg·L^−1^ asparagine, 100 mg·L^−1^ L-pyroglutamic acid, 0.1 mg·L^−1^ indole-3-acetic acid, 1 mg·L^−1^ zeatin riboside, 250 mg·L^−1^ cefotaxime, 100 mg·L^−1^ ticarcillin, 50 mg·L^−1^ vancomycin, and 5 mg·L^−1^ phosphinothricin (PPT), pH 5.6. The explants were transferred to fresh SEM every 2 weeks. During shoot elongation, the elongated shoots were cut using a No. 11 scalpel blade, dipped in 1 mg·L^−1^ indole-3-butyric acid for 3 min, and, then, transferred to RM (rooting medium) containing 4.4 g·L^−1^ MS salt with B5 vitamins, 0.6 g·L^−1^ MES, 3% sucrose, 4.4 g·L^−1^ agar, 25 mg·L^−1^ asparagine, 25 mg·L^−1^ L-pyroglutamic acid, 50 mg·L^−1^ cefotaxime, 50 mg·L^−1^ ticarcillin, and 50 mg·L^−1^ vancomycin, pH 5.6. The rooted plantlets were rinsed with water to wash off the agar medium and transplanted to soil containing a mixture of Biosoil (Hongnong Horticulture, Korea) and vermiculite at a ratio of 3:1 in a magenta jar (SPL, Korea) for 1–3 weeks. T_0_ plants were then transplanted to large pots and grown in a greenhouse. Two trifoliate leaves of the T_0_ plants were screened using an herbicide assay for the identification of putative transformants expressing the *Bar* gene. The herbicide assay was performed on the upper surface of a leaf painted with a mixture of 100 mg·L^−1^ PPT and Tween-20. The herbicide-resistant T_0_ plants were cultivated in a greenhouse, and the seeds harvested (**Figure 1B**).

To identify gene insertions in the soybean transgenic plants, total genomic DNA was extracted. Specifically, DNA extraction using the CTAB (cetyltrimethylammonium bromide) method was conducted with leaf samples from non-transgenic (NT) and transgenic soybean plants (Lipp et al., 1999). Each sample (200 mg) was transferred to a 2-ml sterile reaction tube and 1 ml of CTAB extraction buffer (20 g·L^−1^ CTAB, 1.4 M NaCl, 0.1 M Tris/HCl, 200 mM EDTA) and 1.4 M β-mercaptoethanol were added. The mixture was vortexed and incubated at 65°C for 60 min. The solution was then centrifuged for 10 min at 12,000 × g. The supernatant was transferred to a new 2-ml sterile reaction tube. Ten microliters of RNase A (10 mg·ml^−1^) was added and the mixture was incubated at 37°C for 60 min. The mixture was then extracted with 800 μl of chloroform:isoamyl alcohol (24:1) and centrifuged for 10 min at 12,000 × g. The upper layer was transferred to a new reaction tube. This step was repeated. Isopropanol (0.6 volumes) was added to the upper phase; the mixture was mixed and incubated for 30 min at −20°C. After incubation, the mixture was centrifuged for 10 min at room temperature and the supernatant was discarded. The pellet was washed with 1 ml of 70% ethanol and centrifuged for 5 min at 12,000 × g. The supernatant was discarded, and the pellet was dried at 37°C for 30 min. Then, the dried pellet was dissolved in 100 μl of deionized water and stored at −20°C.

PCR analysis was conducted using KOD FX (TOYOBO, Japan), according to the manufacturer's instructions, and two primer sets designed to amplify specific regions of the *PfFAD3-1* (1,146 bp) and *Bar* (548 bp) genes. The inserted promoters, including β-conglycinin (548 bp), phaseolin (1,543 bp), and CaMV 35S (953 bp) were also amplified. To verify T-DNA insertion into the soybean plant genome, the DNA sequence from the left border (LB) of the *Bar* gene and that from the *PfFAD3-1* gene to the right border (RB) were amplified to represent both ends of the vector (**Table 1**, **Figure 1**). The primers for *Bar,* the promoters, and *PfFAD3-1* were designed to amplify full sequences. The PCR reactions were conducted using a thermal cycler (Takara, Japan) under the following conditions: 95°C for 5 min, followed by 35 cycles at 95°C for 30 s, 50–65°C for 30 s, and 72°C for 30–90 s, with a final extension at 72°C for 10 min.

**Table 1:** Primer sequences used for PCR assays.

Genes	Primer sequence (5ʹ to 3ʹ)
*PfFAD3-1* (MF611845.1)	Forward: ATGGTGGTTGCTATGGACAAACGT Reverse: GTCAGAAGCATAAACGTAGAGATC
*Bar* (X17220.1)	Forward: ATGAGCCCAGAACGACGCCCGGCC Reverse: GGGTCATCAGATTTCGGTGACGGG
**Promoters**	**Primer sequence (5ʹ to 3ʹ)**
β-conglycinin (GU723691.1)	Forward: ATTTGCCGCTATTAATTAATTTGG Reverse: GTTAGTATATCTTAAATTCTTTAA
Phaseolin (J01263.1)	Forward: CATTGTACTCCCAGTATCATTATA Reverse: AGTAGAGTAGTATTGAATATGAGT
CaMV 35S	Forward: ATGAGCCCAGAACGACGCCCGGCC Reverse: GGGTCATCAGATTTCGGTGACGGG
**Borders**	**Primer sequence (5ʹ to 3ʹ)**
left border to *Bar*	Forward: TGGCTGGTGGCAGGATATATTGTG Reverse: AGACAAGCACGGTCAACTTCCGTA
*PfFAD3-1* to right border	Forward: TGGTACAGAGGCAAGGAATGGAGT Reverse: TTAAACTGAAGGCGGGAAACGACA

For Southern blot analysis, 10 µg genomic DNA from NT and transgenic leaf tissues was digested overnight with *Hind*III (Takara, Japan), fractionated on 0.8% agarose gels by electrophoresis, and transferred onto a Hybond N+ nylon membrane (Amersham Pharmacia, USA). Hybridization, washing, and detection were carried out with a digoxigenin (DIG)-labeled DNA probe and chemiluminescence system (Roche, Germany), according to the manufacturer's instructions. The *Bar* primers (5ʹ-AACTTCCGTACCGAGCCGCA-3ʹ/5ʹ-TCGTAGGCGTTGCGTGCCTT-3ʹ) were used to generate the DIG-labeled probe by PCR amplification. Real-time PCR (qPCR) was also performed with NT and transgenic leaf tissues to examine the transgene insertion events, using the CFX-96™ Real-Time System (Bio-Rad, Hercules, CA, USA), following the reaction described by Kim et al. (2016). Each reaction contained 4 μl of 3.3 ng·μl^−1^ DNA, 1 μl of a mixture of 5 pmol·μl^−1^ forward primer (5ʹ-AACTTCCGTACCGAGCCGCA-3ʹ) and 5 pmol·μl^−1^ reverse primer (5ʹ-TCGTAGGCGTTGCGTGCCTT-3ʹ), 5 μl of water, and 10 μl of iQ™SYBR^®^ Green Supermix (Bio-Rad, Ca, USA), to make a total volume of 20 μl. The amplification conditions were as follows: 95°C for 3 min, 40 cycles of 10 s at 95°C, and 30 s at 60°C, and, finally, 95°C for 10 s. To verify amplification specificity, a dissociation curve was generated by increasing the temperature from 65°C–95°C. The *Bar* primers (5ʹ-AACTTCCGTACCGAGCCGCA-3ʹ/5ʹ-TCGTAGGCGTTGCGTGCCTT-3ʹ) were used. A homozygous transgenic plant already confirmed to have a single *Bar* gene introgression was used as a single copy control.

### Analysis of *PfFAD3-1* Gene Expression in Soybean Transformants

Total RNA was isolated from the leaves (T_0_) and seeds (T_2_) of NT and transgenic plants using Plant RNA Purification Reagent (Invitrogen, USA), according to the manufacturer's instructions. One gram of leaves and three seeds were ground using a mortar and pestle with liquid nitrogen and Tris-HCl (pH 9.0), respectively. Each sample (200 mg) was transferred to a 2-ml sterile reaction tube. Subsequently, 1 ml of Plant RNA Purification Reagent was added. The mixture was vortexed and incubated at room temperature for 5 min. The solution was then centrifuged for 10 min at 12,000 × g. The supernatant was transferred to a new 2-ml sterile reaction tube and 150 μl of 5 M NaCl and 450 μl of chloroform were added. The mixture was then vortexed and centrifuged for 10 min at 4°C and 12,000 × g. The upper layer was transferred to a new reaction tube. An equal volume of isopropanol was added and the mixture was incubated at room temperature for 10 min. After incubation, the mixture was centrifuged for 10 min at 4°C and 12,000 × g and the supernatant was discarded. The pellet was washed with 1 ml of 75% ethanol and centrifuged for 3 min at 12,000 × g. The supernatant was discarded and the pellet was dried for 20 min. Then, the dried pellet was dissolved in 20 μl of RNase-free water and stored at −70°C. Reverse transcription PCR (RT-PCR) was performed using the RT-PCR Premix Kit (Genetbio, South Korea), as per the manufacturer's instructions. Primers for the *PfFAD3-1* (1,146 bp product) and *Bar* (548 bp product) genes were used to confirm expression levels (**Table 2**). The constitutively expressed *TUB* gene (256 bp) was used as a reference to normalize the amplified test genes. The PCR reactions were conducted using a thermal cycler (Takara, Japan) under the following conditions: 45°C for 30 min and 95°C for 5 min, followed by 35 cycles at 95°C for 30 s, 59°C–65°C for 30 s, and 72°C for 30–60 s, with a final extension at 72°C for 5 min.

**Table 2:** Primer sequences used for RT-PCR assays.

Genes	Primer sequence (5ʹ to 3ʹ)
*PfFAD3-1* (MF611845.1)	Forward: ATGGTGGTTGCTATGGACAAACGT Reverse: GTCAGAAGCATAAACGTAGAGATC
*Bar* (X17220.1)	Forward: ATGAGCCCAGAACGACGCCCGGCC Reverse: GGGTCATCAGATTTCGGTGACGGG
*TUB*	Forward: TGAGCAGTTCACGGCCATGCT Reverse: CTCGGCAGTGGCATCCTGGT

### Fatty Acid Analysis

Ten milligrams of seed powder obtained by crushing several seeds (n = ~5) with a metal ball in a mill was transferred to a 10-ml glass tube with a Teflon-sealed cap. In total, 0.5 ml toluene and 0.5 ml 5% sulfuric acid (H_2_SO_4_) (v/v) in methanol containing 100 μg of pentadecanoic acid (15:0) as an internal standard were added to each sample. Fatty acids were extracted and transmethylated in a water bath at 90°C for 90 min. Each sample was treated with 1.0 ml of 0.9% sodium chloride (NaCl) solution and 0.5 ml n-hexane and vigorously shaken for extraction. The upper phase containing fatty acid methyl esters (FAMEs) was transferred to a new uncapped glass tube after centrifugation at 2,000 rpm for 2 min. The FAMEs collected after three extractions with 0.5 ml n-hexane were dried with nitrogen gas and dissolved in 0.3 ml n-hexane. The FAMEs were analyzed using a GC-2010 Plus (Shimadzu, Japan) gas chromatograph coupled to a flame ionization detector (FID) and a 30 m × 0.25 mm (inner diameter) HP-FFAP column (Agilent, USA). The oven temperature was increased from 190°C to 232°C at 3°C/min. Nitrogen was used as the carrier gas. The fatty acid content was calculated as the average of three biologically independent sample measurements.

### Analysis of Plant Growth and Phenotype of Soybean Transgenic Plants

NT and transgenic soybean seeds (T_1_) were planted in a seedling tray in June, 2018, and the early leaves were screened by herbicide painting (100 mg·L^−1^ PPT). Herbicide-resistant seedlings were then transplanted into a GMO field (Gunwi, South Korea). Agronomic traits, including plant height; number of branches, nodes, pods, and total seeds; and total seed weight, of the transgenic plants (T_1_) were determined in October, 2018, and compared with those of the NT plants (n = 10 each). To compare the seed size between NT and transgenic soybean seeds, NT and T_2_ soybean seeds harvested from the GMO field were randomly selected and horizontally placed in a row (n = 10 each). The size of the NT and T_2_ seeds was measured from 10 different sets of samples.

### Statistical Analysis of Data

Statistical analysis was performed using the Excel *T* Test program to confirm significant differences between means. Asterisks indicate significant differences compared to NT plants (^*^P < 0.05; ^**^P < 0.01).

## Results

### Production of Transgenic Soybean Plants With Three Types of *PfFAD3-1* Expression Vectors

To produce transgenic soybean plants expressing high ALA levels, the *PfFAD3-1* gene was cloned with two different types of promoters, namely, the seed-specific promoters from soybean β-conglycinin (Pβ-con) and kidney bean phaseolin (Pphas) for soybean seed-specific expression (Sengupta-Gopalan et al., 1985; Doyle et al., 1986) and CaMV 35S (P35S) for constitutive overexpression. The two seed-specific promoters were used to compare the efficiency of each promoter in soybean seed. The corresponding Pβ-con:*PfFAD3-1*, Pphas : *PfFAD3-1* and P35S:*PfFAD3-1* plasmids (**Figure 1A**) were used for *Agrobacterium*-mediated transformation of imbibed mature half seeds of the Korean soybean cultivar “Kwangankong” following the modified half-seed method described by Kim et al. (2013; 2016) (**Figure 1B**). According to the herbicide assay results, the transformation efficiency was approximately 2%, 1%, and 3% with Pβ-con:*PfFAD3-1*, Pphas : *PfFAD3-1*, and P35S:*PfFAD3-1*, respectively. In total, 21, 7, and 17 transgenic plants were produced with Pβ-con:*PfFAD3-1*, Pphas : *PfFAD3-1*, and P35S:*PfFAD3-1*, respectively.

### Confirmation of Introduced Genes in *PfFAD3-1* Transformed Soybeans

**Figure 2:**
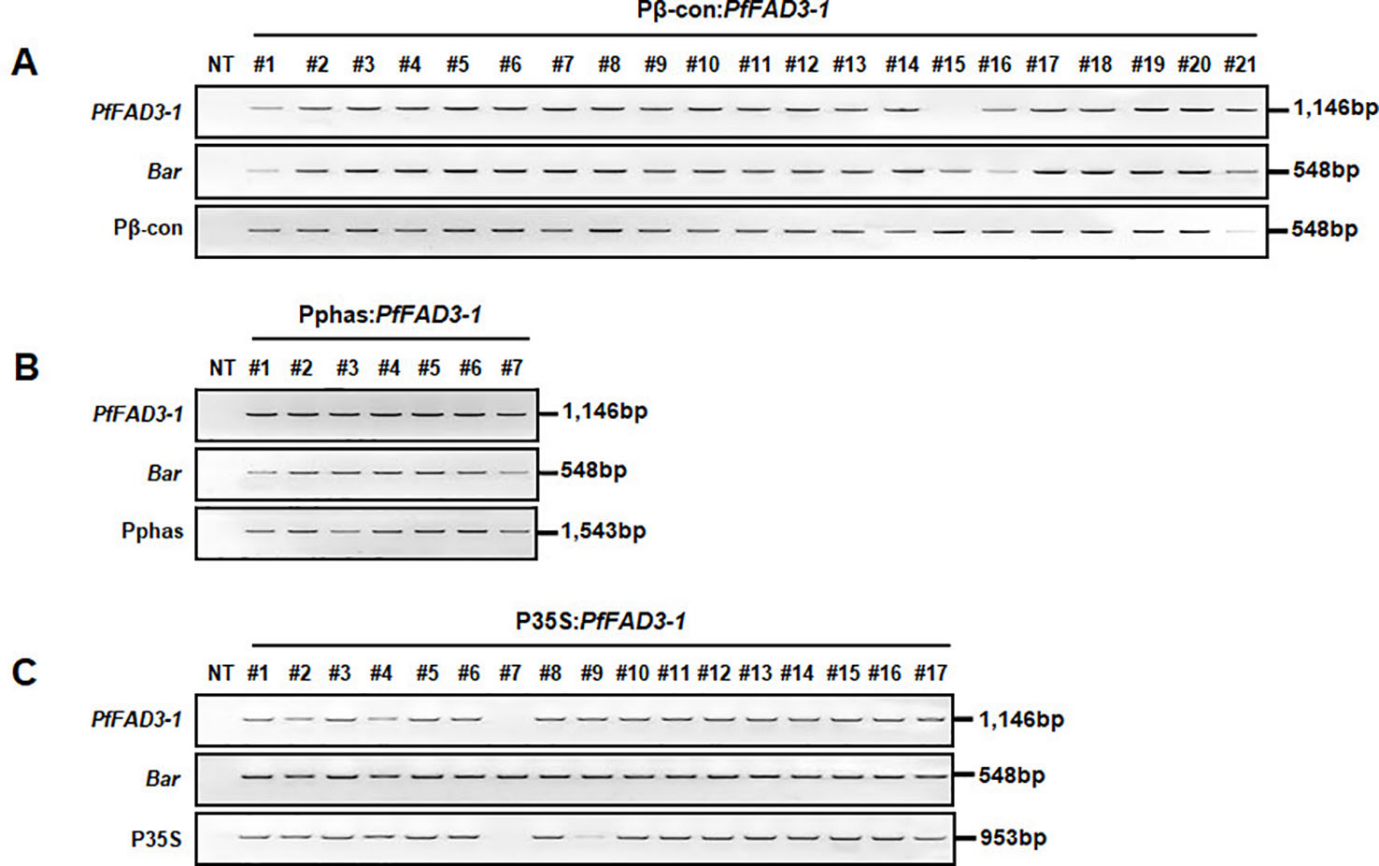
Confirmation of introduced genes from *PfFAD3-1* transformants (T_0_) using PCR. Genomic DNA was extracted from Pβ-con:*PfFAD3-1* **(A)**, Pphas : *PfFAD3-1* **(B)**, and P35S:*PfFAD3-1* **(C)** T_0_ transgenic leaf tissues. *PfFAD3-1*, *PfFAD3-1* gene; *Bar*, *Bar* gene; Pβ-con, β-conglycinin promoter; Pphas, phaseolin promoter; P35S, CaMV 35S promoter; NT, non-transgenic plant; #1–#21 (part A), Pβ-con:*PfFAD3-1* transgenic lines (T_0_); #1–#7 (part B), Pphas : *PfFAD3-1* transgenic lines (T_0_); #1–#17 (part C), P35S:*PfFAD3-1* transgenic lines (T_0_).

Leaf tissues from the 21 Pβ-con:*PfFAD3-1*, 7 Pphas : *PfFAD3-1*, and 17 P35S:*PfFAD3-1* transgenic plants (T_0_) were used to confirm the integration of the transgene with PCR using *PfFAD3-1* and *Bar* primers that amplified 1,146 and 548 bp DNA fragments, respectively. In addition, the DNA regions of the β-conglycinin, phaseolin, and CaMV 35S promoters were amplified as 548, 1,543, and 953 bp fragments, respectively (**Figure 2**). The results of the PCR analysis for the 21 Pβ-con:*PfFAD3-1* transgenic lines confirmed the insertion of the transgene and β-conglycinin promoter sequence in all lines except line #15 (missing a region of the *PfFAD3-1* gene) (**Figure 2A**). All seven putative Pphas : *PfFAD3-1* transgenic plants successfully represented the transgene and phaseolin promoter (**Figure 2B**). Among the 17 P35S:*PfFAD3-1* transgenic lines, only line #7 was missing regions of both the *PfFAD3-1* gene and CaMV 35S promoter (**Figure 2C**). The selectable marker (*Bar* gene) was introduced in all *PfFAD3-1-*transformed soybean plants. Moreover, T-DNA insertion was verified by the amplification of both end regions of the vector construct (data not shown).

**Figure 3:**
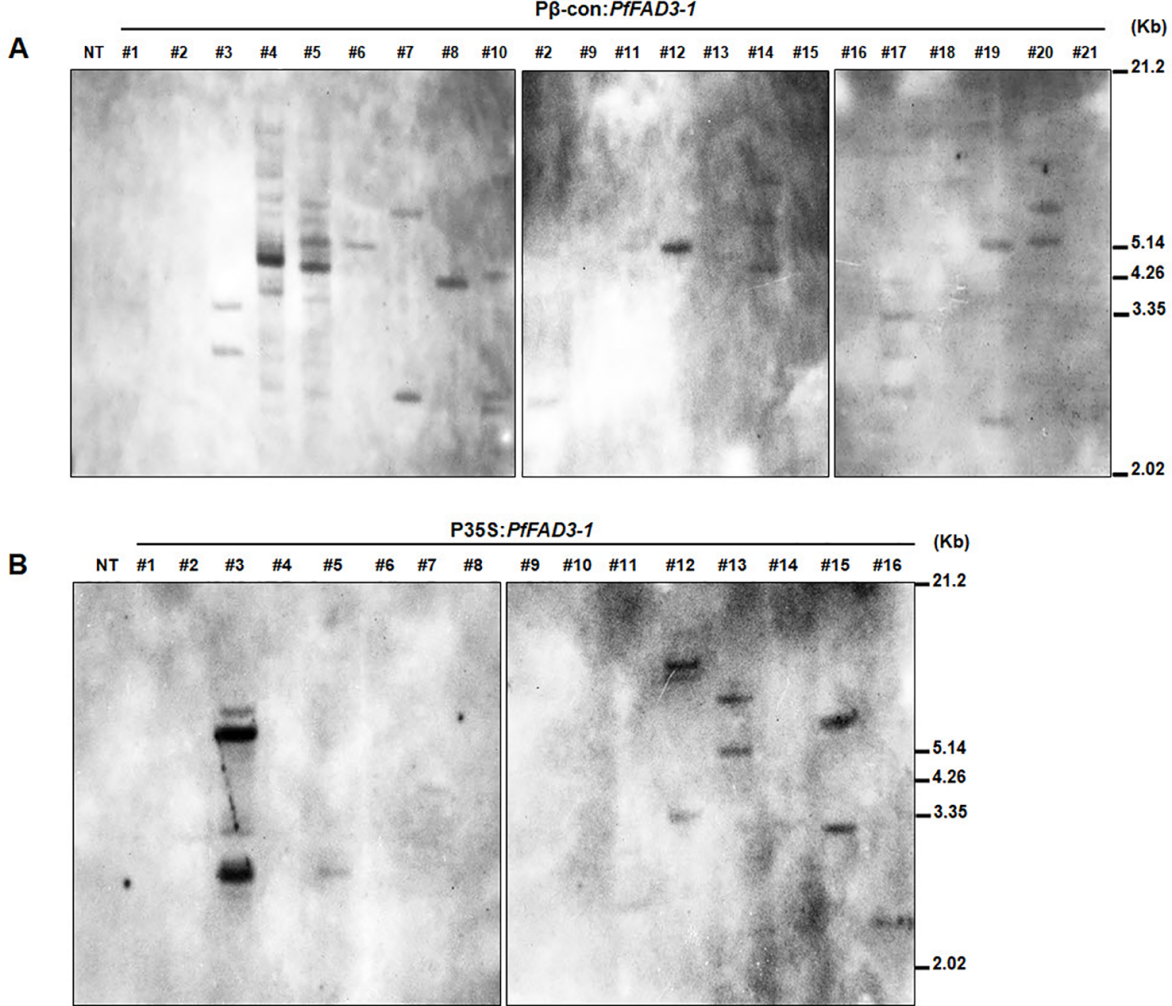
Genomic Southern blot analysis of *PfFAD3-1* transgenic soybean (T_0_). Ten micrograms of genomic DNA from Pβ-con:*PfFAD3-1* **(A)** and P35S:*PfFAD3-1* **(B)** transgenic plants (T_0_) were digested with *Hind*III and hybridized with the *Bar* probe. Approximate DNA size markers are indicated on the *right*. NT, non-transgenic plant; #1–#21 (part A), Pβ-con:*PfFAD3-1* transgenic lines (T_0_); #1–#16 (part B), P35S:*PfFAD3-1* transgenic lines (T_0_).

We determined the copy numbers of transgene insertions by performing genomic Southern blots of the 21 Pβ-con:*PfFAD3-1* and 16 P35S:*PfFAD3-1* transgenic plants (T_0_) (**Figure 3**). Hybridization with a *Bar* probe revealed that Pβ-con:*PfFAD3-1* transgenic lines #6, #8, #11, and #12 had single insertions and lines #2, #3, #4, #5, #7, #10, #14, #17, #19, and #20 contained multiple copies (**Figure 3A**). Integration of the transgene in the P35S:*PfFAD3-1* transgenic plants (except line #17, which completely withered) was also confirmed, indicating a low copy number of the transgene in lines #5, #7, #11, and #16 and multiple insertions in lines #3, #12, #13, and #15 (**Figure 3B**). Given the limited number of plants and leaf tissue samples of the Pphas : *PfFAD3-1* transformants, their copy numbers were estimated by qPCR of the *Bar* gene, instead of Southern blot analysis (**Supplementary Figure 1**). Most transgenic lines of the Pphas : *PfFAD3-1* transformants possessed low insert copy numbers, except line #2. Considering the homozygous single copy control of the *Bar* gene, most transgenic lines contained a single copy.

### Transgene Expression in *PfFAD3-1* Transformed Soybeans

**Figure 4:**
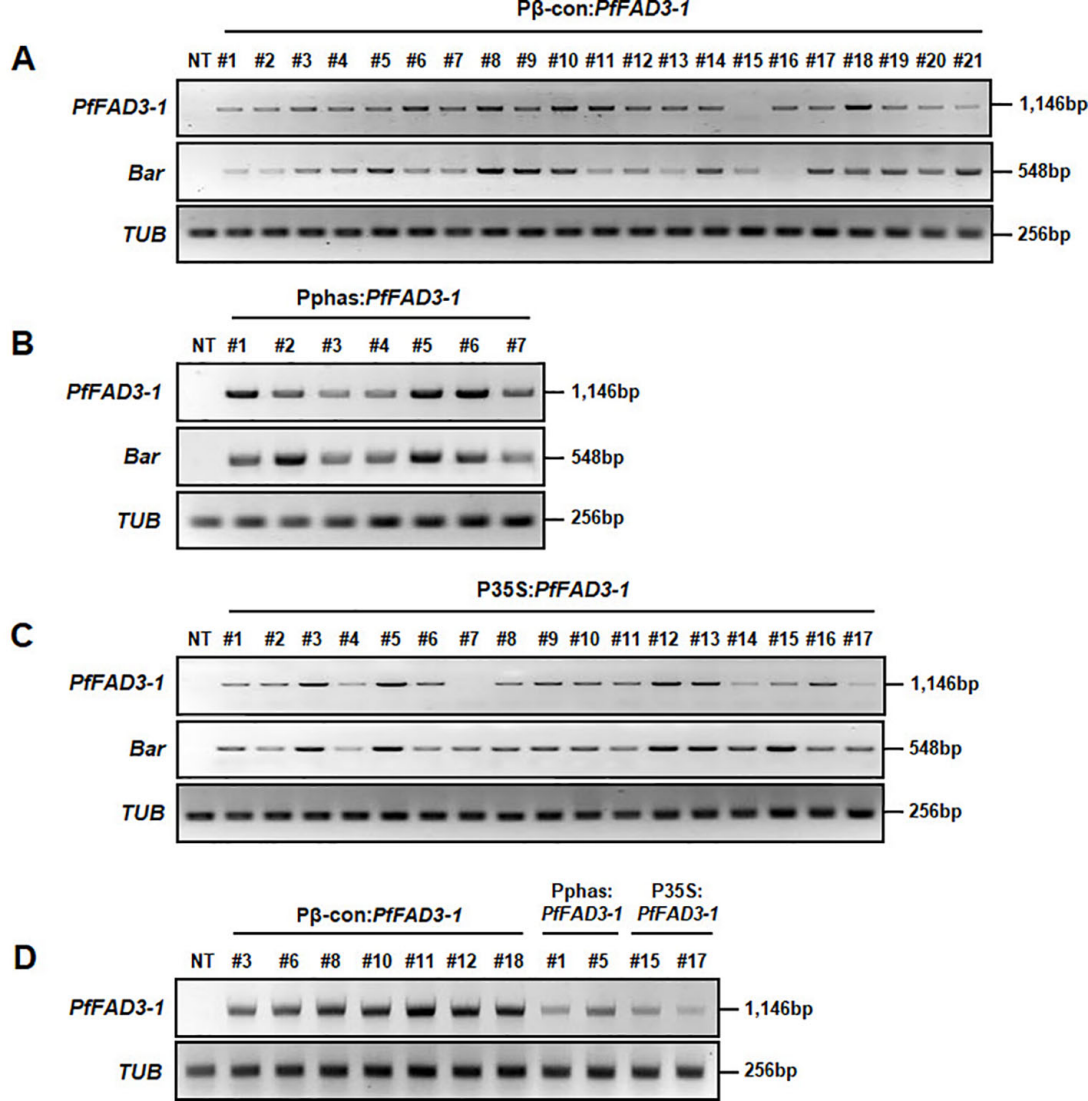
Transcript levels of *PfFAD3-1* and *Bar* genes in transgenic plants (T_0_) and seeds (T_2_) using reverse transcriptase PCR (RT-PCR). Total RNA was extracted from Pβ-con:*PfFAD3-1* **(A)**, Pphas : *PfFAD3-1* **(B)**, and P35S:*PfFAD3-1* **(C)** T_0_ transgenic leaf tissues and from T_2_ seeds **(D)**. The *TUB* gene was used as a quantitative control. NT, non-transgenic plant; #1–#21 (part A), Pβ-con:*PfFAD3-1* transgenic lines (T_0_); #1–#7 (part B), Pphas : *PfFAD3-1* transgenic lines (T_0_); #1–#17 (part C), P35S:*PfFAD3-1* transgenic lines (T_0_); #3, #6, #8, #11, #12, and #18, T_2_ seeds of Pβ-con:*PfFAD3-1* transgenic lines; #1 and #5, T_2_ seeds of Pphas : *PfFAD3-1* transgenic lines; #15 and #17, T_2_ seeds of P35S:*PfFAD3-1* transgenic lines (part D).

The expression levels of the *PfFAD3-1* and *Bar* genes were analyzed in the Pβ-con:*PfFAD3-1*, Pphas : *PfFAD3-1* and P35S:*PfFAD3-1* transgenic plants (T_0_) using RT-PCR (**Figure 4**). The results from the 21 Pβ-con:*PfFAD3-1* transgenic lines confirmed the expression of the *PfFAD3-1* gene in all lines except line #15. The relative level of expression was remarkably higher in lines #6, #8, #10, #11, and #18 than in the other transgenic lines (**Figure 4A**). Expression of the *PfFAD3-1* gene was confirmed in all seven Pphas : *PfFAD3-1* transgenic lines. Of these, lines #1, #5, and #6 showed noticeably high expression of the *PfFAD3-1* gene (**Figure 4B**). The integrated *PfFAD3-1* gene was expressed in all 17 P35S:*PfFAD3-1* transgenic lines as expected, except line #7 (**Figure 4C**). Expression of the selectable marker *Bar* was confirmed in all *PfFAD3-1*-transformed soybean plants except Pβ-con:*PfFAD3-1* line #16. In addition, the *PfFAD3-1* and *Bar* genes were not detected in the NT plants. Using RT-PCR, the expression level of the *PfFAD3-1* gene was also analyzed in Pβ-con:*PfFAD3-1* (lines #3, #6, #8, #10, #11, #12, and #18), Pphas : *PfFAD3-1* (lines #1 and #5), and P35S:*PfFAD3-1* (lines #15 and #17) transgenic seeds (T_2_) with higher 18:3 content (**Figure 4D**). *PfFAD3-1* expression was much higher with the seed-specific β-conglycinin promoter (Pβ-con:*PfFAD3-1*) than with the seed-specific phaseolin promoter (Pphas : *PfFAD3-1*) and 35S promoter (P35S:*PfFAD3-1*). Expression from the seed-specific phaseolin promoter (Pphas : *PfFAD3-1*) was higher than or similar to the 35S promoter (P35S:*PfFAD3-1*).

### Seed Fatty Acids in Soybean Transformants

**Figure 5:**
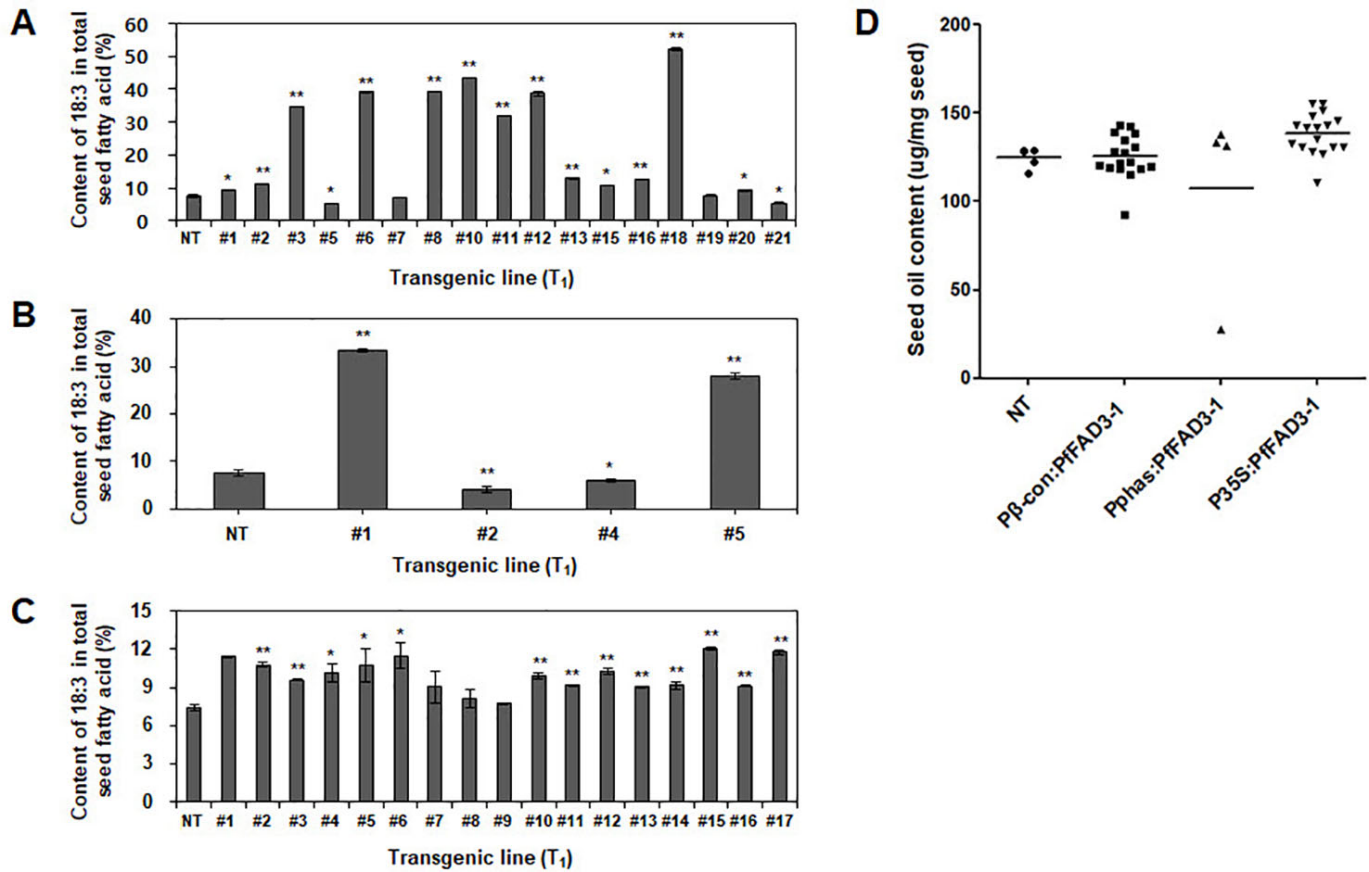
Content of α-linolenic acid (ALA) and seed oil in transgenic soybean seeds (T_1_). Content of ALA in Pβ-con:*PfFAD3-1* **(A)**, Pphas : *PfFAD3-1* **(B)**, and P35S:*PfFAD3-1* **(C)** was analyzed using gas chromatography. **(D)** Seed oil content analysis of three *PfFAD3-1* transgenic soybeans. NT, non-transgenic plant; #1–#21 (part A), T_1_ seeds of Pβ-con:*PfFAD3-1* transgenic lines; #1–#5 (part B), T_1_ seeds of Pphas : *PfFAD3-1* transgenic lines; #1–#17 (part C), T_1_ seeds of P35S:*PfFAD3-1* transgenic lines. In parts A–C, error bars indicate the means ± standard deviations; asterisks indicate significant changes compared to NT values (* = *p* < 0.05; ** = *p* < 0.01). In part D, dots represent the seed oil content from each line (triplicate measurements); bars indicate mean values.

The fatty acid composition of the soybean seeds was analyzed using gas chromatography to determine the change in seed fatty acid composition upon heterologous *PfFAD3-1* expression. The 18:3 content was 7.5% in the NT soybean “Kwangankong” and increased up to 52.4% in Pβ-con:*PfFAD3-1*, a 7-fold increase (**Figure 5A**, **Table 3**). In the case of Pphas : *PfFAD3-1*, the content increased up to 33.3%, which was 4.4 times the level reported for “Kwangankong” (**Figure 5B**, **Table 4**). However, the 18:3 content increased up to 12.1% in P35S:*PfFAD3-1*, an increase of only 1.6 fold (**Figure 5C**, **Table 4**).

**Table 3:** Fatty acid composition of Pβ-con:*PfFAD3-1* transgenic soybeans (T_1_ seeds).

FA	NT (n = 4)	Pβ-con:*PfFAD3-1*																	
	Mean	#1	#2	#3	#5	#6	#7	#8	#10	#11	#12	#13	#15	#16	#18	#19	#20	#21	Mean
16:0	12.8	14.5	15.4	15.6	13.5	15.6	15.2	15.2	16.0	16.2	16.6	16.3	15.2	16.0	15.7	16.3	16.3	15.2	15.9
18:0	3.8	4.6	4.1	3.9	4.2	3.8	3.6	3.9	3.9	3.8	4.1	4.0	4.1	4.1	4.4	3.8	4.0	4.4	4.1
18:1	37.0	27.2	22.1	20.0	41.1	19.5	26.7	22.1	18.4	18.0	19.5	20.2	23.1	19.0	21.1	23.8	21.3	29.7	24.0
18:2	38.9	44.2	47.0	25.9	36.1	22.1	47.5	19.6	18.2	30.0	21.1	46.6	46.9	48.1	6.2	48.3	49.2	45.2	37.2
18:3	7.5	9.4	11.4	34.5	5.1	39.1	7.1	39.2	43.5	32.0	38.6	12.9	10.7	12.8	52.6	7.8	9.2	5.5	18.8

NT represents non-transformant wild-type (Kwangankong). Fatty acids below 1% were omitted. The data are mean value by three independent experiments and the unit of value is mole%.

**Table 4:** Fatty acid composition of Pphas : *PfFAD3-1* and P35S:*PfFAD3-1* transgenic soybeans (T_1_ seeds).

FA	NT (n = 4)	Pphas : *PfFAD3-1*					P35S:*PfFAD3-1*																	
	Mean	#1	#2	#4	#5	Mean	#1	#2	#3	#4	#5	#6	#7	#8	#9	#10	#11	#12	#13	#14	#15	#16	#17	Mean
16:0	12.8	14.3	34.2	16.2	15.6	20.1	15.6	16.7	15.8	15.7	16.8	13.5	16.0	13.1	11.7	12.5	16.9	15.8	15.5	15.2	16.1	15.3	15.7	15.6
18:0	3.8	4.6	9.4	4.4	4.0	5.6	4.3	4.4	4.2	4.6	4.3	4.5	4.5	4.4	4.3	4.6	4.9	4.4	4.4	4.4	4.4	4.8	4.2	4.5
18:1	37.0	24.2	37.6	26.7	26.3	28.7	19.8	20.0	21.8	23.0	18.6	37.2	23.7	42.5	51.7	46.3	21.9	22.6	26.1	28.1	20.4	22.9	20.0	22.8
18:2	38.9	23.4	14.7	46.7	26.0	27.7	48.7	48.0	48.6	46.5	49.5	33.3	46.7	31.9	24.6	26.6	46.9	46.9	45.0	43.0	47.0	47.7	48.3	46.5
18:3	7.5	33.4	4.1	6.0	28.1	17.9	11.5	10.9	9.6	10.2	10.8	11.5	9.1	8.2	7.8	10.0	9.3	10.3	9.1	9.2	12.1	9.2	11.8	10.6

NT represents non-transformant wild-type (Kwangankong). Fatty acids below 1% were omitted. The data are mean value by three independent experiments and the unit of value is mole%.

**Figure 6:**
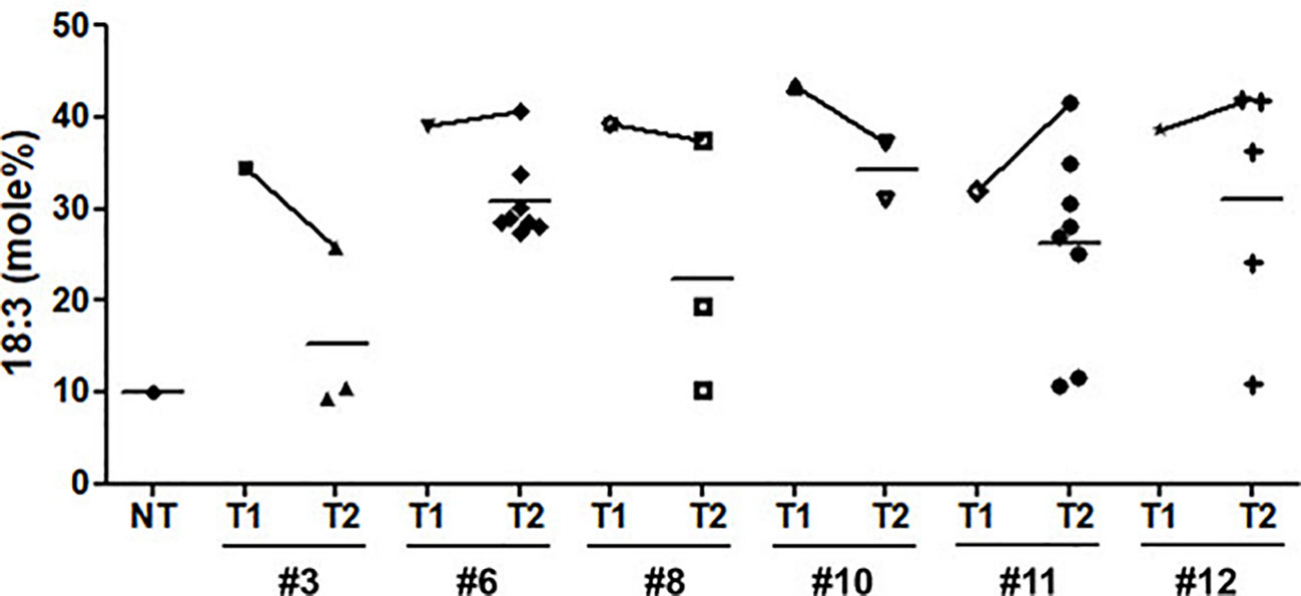
Comparison of α-linolenic acid (ALA) content between T_1_ and T_2_ seeds of Pβ-con:*PfFAD3-1* transgenic soybeans. NT, non-transgenic. Symbols represent the ALA content of each line performed in triplicate. Horizontal bars indicate mean values.

Seed oil content in the three types of transgenic soybean were analyzed and compared with that in wild-type plants. The average seed oil content for Pβ-con:*PfFAD3-1*, Pphas : *PfFAD3-1*, and P35S:*PfFAD3-1* soybean was 125.4, 107.5, and 138.3 μg/mg seed, respectively (**Figure 5D**). For wild-type plants, the average seed oil content was 124.7 μg/mg seed. No statistically significant difference was observed in the seed oil content between transgenic and wild-type soybeans by one-way analysis of variance (ANOVA). In all *PfFAD3-1* transgenic soybeans, the content of saturated fatty acid increased by 1%–5% and that of 18:1 and 18:2 decreased; the content of 18:3 increased compared with that in the wild-type soybean. In particular, 38% or less increase in the content of 18:3 in the Pβ-con:*PfFAD3-1* soybean resulted in a larger decrease in 18:1 content than 18:2 content. Meanwhile, a >38% increase in the content of 18:3 induced a larger decrease in 18:2 content than 18:1 content (**Table 3**). For the Pphas : *PfFAD3-1* soybean, the 18:2 content decreased more than the 18:1 content, with a rise in 18:3 content to ≥28%. The content of 18:3 slightly increased in the P35S:*PfFAD3-1* soybeans compared with that in the wild-type plants and reached a maximum of 4.6%. Regardless of the magnitude of increase in 18:3 content, an inverse relationship was observed between 18:1 and 18:2 content (**Tables 3** and **4**). Furthermore, the increase in fatty acid content was maintained in the T_2_ generation. The 18:3 content of the Pβ-con:*PfFAD3-1* T_2_ seeds increased up to 42%, showing a 4.2-fold rise compared with that in the wild-type “Kwangankong” (**Figure 6**, **Table 5**). Unlike the fatty acid composition of the Pβ-con:*PfFAD3-1* T_1_ seeds, the saturated fatty acid content decreased by 3%–5% in T_2_ compared with that in the wild-type soybean (**Table 5**). A comparison of the fatty acid compositions of the T_1_ and T_2_ seeds revealed an average decrease of 4.6% in the saturated fatty acid content in T_2_ (**Tables 3** and **5**). The decrease in 18:1 content in T_2_ was not significant. One transgenic line (#11–7) had a 3.7% increase in 18:1 content, although the 18:3 content was greater than 30%.

**Table 5:** Fatty acid composition of Pβ-con:*PfFAD3-1* soybeans (T_2_ seeds).

FA	NT	#3			#6								#8			#10		#11								#12				
		−1	−2	−3	−1	−2	−3	−5	−6	−7	−8	−9	−1	−2	−3	−1	−2	−1	−2	−3	−4	−5	−6	−7	−8	−1	−2	−3	−4	−5
16:0	14.2	11.8	11.7	12.5	11.9	11.9	12.1	11.8	12.2	12.1	11.3	11.1	11.7	11.7	11.2	11.4	11.6	13.3	11.9	11.9	12.5	11.9	12.3	11.5	11.7	11.9	11.4	12.9	12.2	12.6
18:0	6.7	4.7	4.5	5.2	4.7	5.1	4.7	4.8	4.9	5.1	4.9	4.9	4.8	5.1	4.4	4.1	4.7	4.6	4.2	4.7	4.8	4.7	4.6	4.4	4.3	5.0	4.7	4.5	4.8	4.8
18:1	32.2	35.8	34.5	33.4	30.5	29.0	27.5	31.1	26.7	29.0	32.5	35.2	34.7	32.6	34.3	33.9	33.5	27.7	33.3	31.4	29.7	31.7	30.6	36.0	31.3	27.7	32.7	26.9	28.2	28.7
18:2	37.0	37.2	23.6	39.6	24.4	20.2	27.5	23.8	27.2	13.2	21.1	21.3	29.4	40.3	12.7	13.3	19.2	43.6	25.6	23.9	41.4	21.1	25.6	13.2	11.1	13.5	15.0	31.5	13.0	42.9
18:3	9.9	10.5	25.8	9.4	28.5	33.9	28.2	28.5	29.0	40.7	30.2	27.5	19.4	10.3	37.4	37.3	31.0	10.8	25.1	28.0	11.7	30.5	26.9	34.9	41.6	42.0	36.2	24.2	41.8	11.0

NT represents non-transformant wild-type (Kwangankong). Fatty acids below 1% were omitted. The data are mean value by three independent experiments and the unit of value is mole%.

### Yield Components and Seed Phenotypes of Pβ-con:*PfFAD3-1* Transformed Soybeans

**Figure 7:**
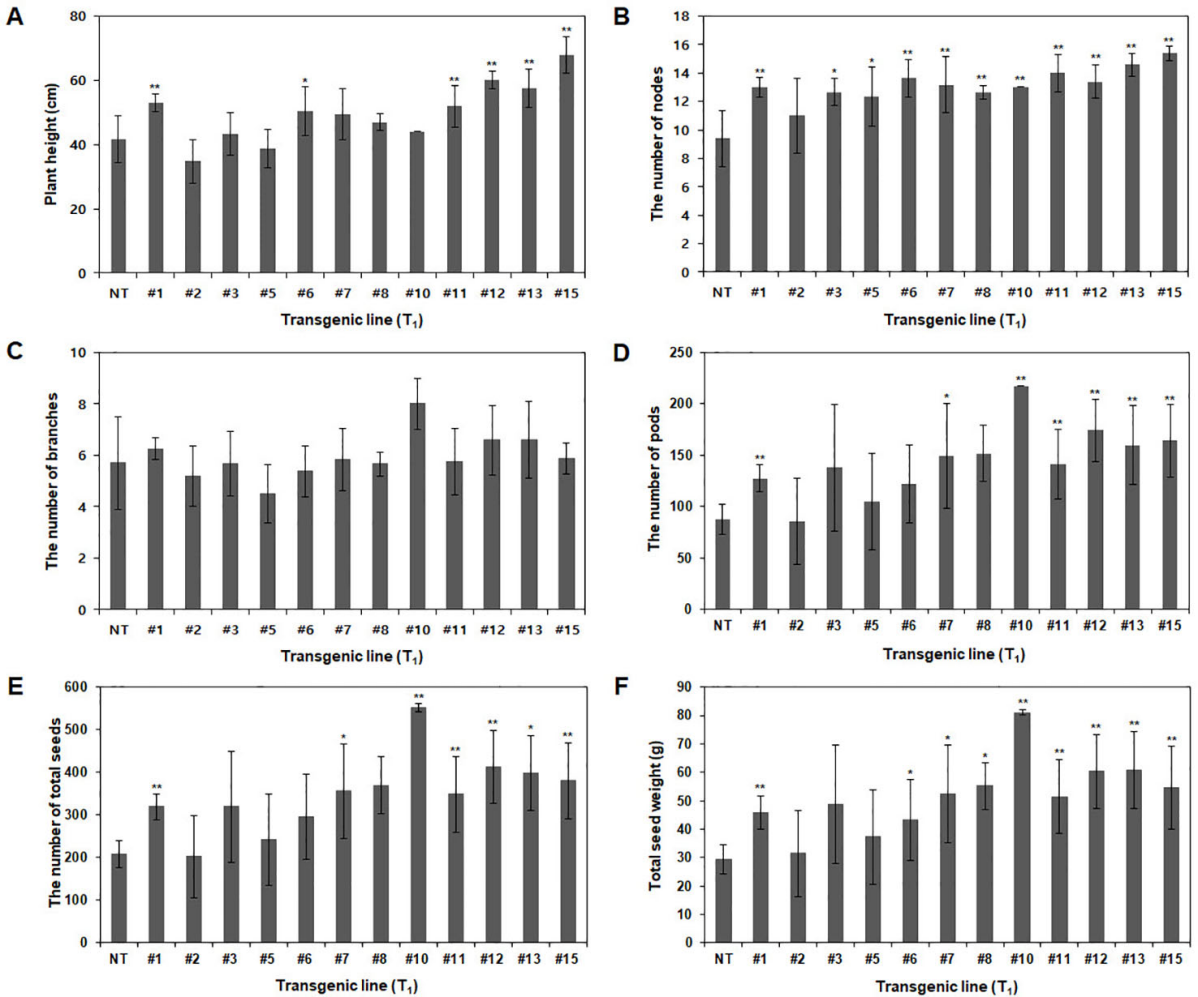
Agronomic characteristics of Pβ-con:*PfFAD3-1* transgenic plants (T_1_) in a GMO field. Non-transgenic and T_1_ Pβ-con:*PfFAD3-1* transgenic plants were cultivated in a GMO field, and agronomic traits such as plant height **(A)**, number of nodes **(B)**, number of branches **(C)**, number of pods **(D)**, total number of seeds **(E)** and total seed weight **(F)** were investigated. NT, non-transgenic plants; #1–#15, Pβ-con:*PfFAD3-1* transgenic lines (T_1_). Data represent the means (± SD) from ten independent biological replicates for each line. Asterisks indicate significant changes compared to NT values (* = *p* < 0.05; ** = *p* < 0.01).

**Figure 8:**
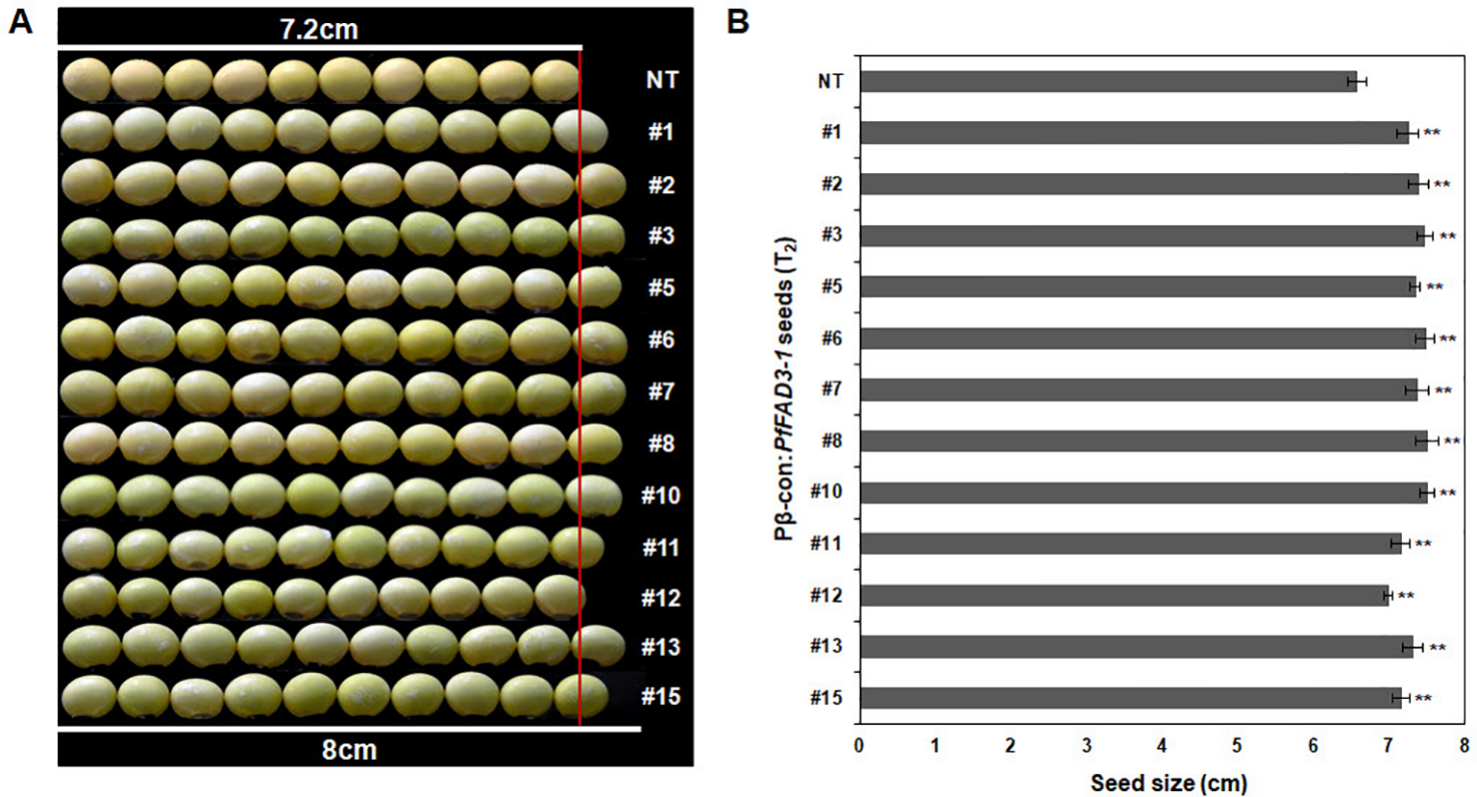
Comparison of sizes of T_2_ seeds from Pβ-con:*PfFAD3-1* transgenic lines and non-transgenic soybean seeds. **(A)** Comparison of seed size between non-transgenic and T_2_ Pβ-con:*PfFAD3-1* soybean seeds. Non-transgenic and T_2_ Pβ-con:*PfFAD3-1* soybean seeds were randomly chosen and horizontally placed in a row (*n* = 10 each). **(B)** Measurement of soybean seed size. The size of non-transgenic and T_2_ Pβ-con:*PfFAD3-1* soybean seeds was measured ten times. NT, non-transgenic soybean seeds; #1–#15, T_2_ Pβ-con:*PfFAD3-1* soybean seeds. Bars indicate the means ± standard deviations. Asterisks indicate significant changes compared to NT values (** = *p* < 0.01).

Agronomic traits, including plant height, number of branches, nodes, pods, and total seeds and total seed weight, were investigated in the GMO field (Gunwi, South Korea) from the 12 Pβ-con:*PfFAD3-1* transgenic lines (T_1_) (**Figure 7**). The heights of the transgenic lines #1, #6, #11, #12, #13, and #15 were significantly higher than those of the NT plants (*p* < 0.05 for line #6 and *p* < 0.01 for lines #1, #11, #12, #13, and #15) (**Figure 7A**). The transgenic lines had more nodes but similar or higher numbers of branches than the NT plants (**Figures 7B, C**). The number of pods was higher in the transgenic lines than in the NT plants, representing a significant increase of approximately 45%, 70%, 147%, 61%, 99%, 82%, and 87% in lines #1, #7, #10, #11, #12, #13, and #15, respectively (*p* < 0.05 for line #7 and *p* < 0.01 for lines #1, #10, #11, #12, #13, and #15) (**Figure 7D**). The total number of seeds and total seed weight, which exerts a decisive influence on yield, were investigated from the 12 Pβ-con:*PfFAD3-1* transgenic lines (T_1_). The total number of seeds from transgenic lines #1, #7, #10, #11, #12, #13, and #15 increased by approximately 54%, 72%, 167%, 69%, 100%, 93%, and 84%, respectively (*p* < 0.05 for lines #7 and #13 and *p* < 0.01 for lines #1, #10, #11, #12, and #15) (**Figure 7E**). The total seed weights of transgenic lines #1, #6, #7, #8, #10, #11, #12, #13, and #15 were approximately 57%, 48%, 78%, 88%, 176%, 75%, 106%, 108%, and 86% higher than those of the NT plants (*p* < 0.05 for lines #6, #7, and #8 and *p* < 0.01 for lines #1, #10, #11, #12, #13, and #15) (**Figure 7F**). Phenotypic observations of the dried seeds indicated an increase in seed size upon expression of the *PfFAD3-1* gene with the β-conglycinin promoter (**Figure 8**). Ten randomly selected Pβ-con:*PfFAD3-1* T_2_ seeds were arranged in a row, and the average length was measured using 10 repeats. As a result, the seed length of the Pβ-*PfFAD3-1*-transformed soybean was 6%–14% higher than that of the wild-type soybean seeds. In particular, the highest increase (14%) among the 12 transgenic lines was observed for lines #3, #6, #8, and #10. In summary, the Pβ-con:*PfFAD3-1* transgenic soybeans had higher yields than the wild-type soybeans and the increase in seed size contributed to the increased yield by affecting the total seed weight.

## Discussion

In this study, we successfully transformed soybean with *PfFAD3-1* and developed a new soybean transformant that produces up to 42% ALA in seed oil. Soybean transformation is based on the cotyledonary node (CN) method (Hinchee et al., 1988) and can be improved by various methods. One of these methods involves the use of half-seed explants (Paz et al., 2006). Since the introduction of the half-seed method, we have utilized this efficient transformation method and overcome many difficulties, such as efficiency, reproducibility, and genotype dependency. With this relatively new and modified transformation protocol, various soybean transgenic plants expressing agronomically important genes, such as those imparting tolerance to drought and salt stress, affording resistance to soybean mosaic virus, expressing high content of secondary metabolites, and exhibiting better yields, have been produced (Jiang et al., 2010; Kim et al., 2012; Kim et al., 2013; Kim et al., 2016; Kim et al., 2017; Kwon et al., 2017; Kim et al., 2018; Cho et al., 2019; Park et al., 2019).

Based on our modified *Agrobacterium*-mediated transformation protocol, the *PfFAD3-1* gene was transformed into soybean to increase the ALA content in the seeds. Soybean seed oil is mainly used for frying in the United States and Europe. Therefore, soybeans containing low amounts of polyunsaturated fatty acids, LA and ALA, which are easily oxidized, and high oleic acid content, which is stable to oxidation at high temperatures, have been developed using genetic engineering or mutant breeding methods (Buhr et al., 2002; Graef et al., 2009; Pham et al., 2010; Pham et al., 2012). The genome editing method with clustered regularly interspaced short palindromic repeats (CRISPR)-Cas9 was recently used to knockout the soybean *FAD2*-2 gene to achieve better production of oleic acid (Al Amin et al., 2019). However, soybean is used as a health functional food in the form of soymilk in Korea and East Asia. To improve the oil components of the seeds, transgenic plants with increased content of omega-3 fatty acids were produced in the present study. ALA is the longest carbon omega-3 fatty acid produced by plants and serves as an essential fatty acid consumed in food. ALA is converted to EPA and DHA by the human body and is involved in metabolism. The soybean genome contains four copies of *FAD3*, three of which are expressed in developing seeds (Bilyeu et al., 2003; Anai et al., 2005). A single copy of *FAD3* is expressed in *Arabidopsis*, which synthesizes 20% of the ALA in the seeds. Perilla (*Perilla frutescens* L.) has two copies of *FAD3*, which synthesize 60% of the ALA in the seeds (Arondel et al., 1992; Lee et al., 2016; Lee et al., 2019). Despite the presence of a higher copy number of *FAD3* in soybean than in other plants, the ALA content in soybean seeds is as low as 8%. Thus, the *FAD3* genes in soybean have weak activity at the transcriptional or translational level or their expression is weak in embryos and endosperms, the sites of oil accumulation. As *PfFAD3-1* isolated from lesquerella showed efficient conversion of LA to ALA in *Arabidopsis* (Lee et al., 2019), the transformation of *PfFAD3-1* to soybean was also expected to enhance ALA content. *PfFAD3* was expressed in soybean using two seed-specific promoters and the 35S promoter. As expected, *PfFAD3* expression under the control of the three promoters resulted in an increase in ALA content in the soybean seeds compared with that in the NT controls. The β-conglycinin and phaseolin promoters showed higher synthesis of ALA than the 35S promoter (**Figure 5**). Thus, the seed-specific promoters induced more *PfFAD3* expression during seed development than the 35S constitutive promoter (**Figure 4D**). Among the two seed-specific promoters, β-conglycinin showed a higher synthesis of ALA than phaseolin (**Figure 5**). Being derived from the same species, β-conglycinin may be more favorable for the transcriptional activity of *PfFAD*3 in soybean than phaseolin from kidney bean. Another possibility is that the regulation of *PfFAD3* expression by β-conglycinin may be more consistent with soybean ALA biosynthesis than the regulation of *PfFAD3* expression by phaseolin.

The activity of the *PfFAD3* transgene was well maintained over generations. ALA content increased by up to 42% in the seeds of the T_2_ generation plants that were considered to be homozygous for the transgene (**Figure 6**). This ALA content is 45% that of flax (*Linum usitatissimum* L.) and 60% that of perilla, the two oil crops with the highest ALA content (Rao et al., 2008). Considering the cultivation area and production of seed oil compared with other oil crops, high omega-3 soybeans that produce 42% ALA may provide a great opportunity for increased omega-3 production.

During the investigation of agronomic traits, such as plant height, number of branches, nodes, pods, and total seeds, and total seed weight, several Pβ-con:*PfFAD3-1* transgenic lines (T_1_) showed a significant increase in most traits (**Figure 7A**). In particular, the number of pods and total seeds and the total seed weight significantly increased. This increase in yield parameters could reflect the apparent increase in the size of seeds from the Pβ-con:*PfFAD3-1* transgenic lines (**Figure 8**). The seed length of Pβ-*PfFAD3-1*-transformed soybeans (lines #3, #6, #8, and #10) increased by more than 10% compared to that of wild-type soybean plants. Further detailed analysis of the seeds is currently underway and we have confirmed an approximately 10% increase in cell number and area (data not shown). Currently, we do not know how the high ALA content of the Pβ-con:*PfFAD3* transformants increases seed yield and seed size. ALA is a precursor to the jasmonic acid (JA) phytohormone. It has been reported that JA treatment increases the grain yield of amaranth in the absence of drought stress (Délano-Frier et al., 2004). ALA treatment of *Arabidopsis* cell suspension culture has been shown to induce the expression of methionine sulfoxide reductase and alkenal reductase genes that protect against abiotic and oxidative stresses (Mata-Pérez et al., 2015). Therefore, it is possible to increase the expression of genes that offer resistance to abiotic stress to increase adaptability to the growing environment or increase growth by inducing resistance to oxidative stress even under normal growth conditions. Investigating the various metabolic changes in plants with an increased ALA content will help to determine the mechanism of increased yields.

## Data Availability Statement

All datasets generated for this study are included in the article/**Supplementary Material**.

## Author Contributions

WY, HJK, K-RL, HC, SJ, and J-YK performed the experiments. HJ and S-WO analyzed the data. HUK and Y-SC wrote the paper. All authors read and approved the final manuscript.

## Funding

This work was supported by a grant from the Next-Generation BioGreen 21 Program, (Project No. PJ01366501), Rural Development Administration, South Korea.

## Conflict of Interest

The authors declare that the research was conducted in the absence of any commercial or financial relationships that could be construed as a potential conflict of interest.

## Abbreviations

ALA: α-linolenic acid (18:3), FAME: fatty acid methyl ester, NT: non-transgenic, CN: cotyledonary node, Pβ: β-conglycinin promoter, Pphas: phaseolin promoter

## Supplementary Material

Supplementary Figure 1 Determination of transgene copy number in Pphas : *PfFAD3-1* transgenic plants with real-time PCR. Genomic DNA was extracted from Pphas : *PfFAD3-1* transgenic plants (T_0_) to determine transgene copy number. *Bar*, target *Bar* gene; Con, homozygous single copy transgenic plant; NT, non-transgenic plant; #1–#7, Pphas : *PfFAD3-1* transgenic lines (T_0_).
