# Supplementary figures and images for: Increased Production of α-Linolenic Acid in Soybean Seeds by Overexpression of Lesquerella FAD3-1
Source: Front Plant Sci. 2020 Jan 31;10:1812. doi: 10.3389/fpls.2019.01812 (PMC7005135; doi:10.3389/fpls.2019.01812)

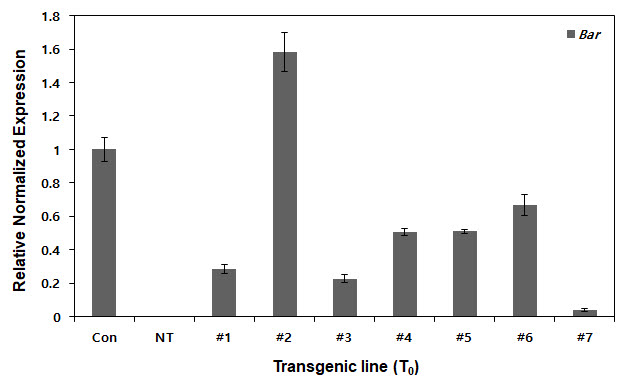

Supplement: Supplementary Figure 1 — Determination of transgene copy number in Pphas : PfFAD3-1 transgenic plants with real-time PCR. Genomic DNA was extracted from Pphas : PfFAD3-1 transgenic plants (T0) to determine transgene copy number. Bar, target Bar gene; Con, homozygous single copy transgenic plant; NT, non-transgenic plant; #1–#7, Pphas : PfFAD3-1 transgenic lines (T0). [file Image_1.jpeg]
